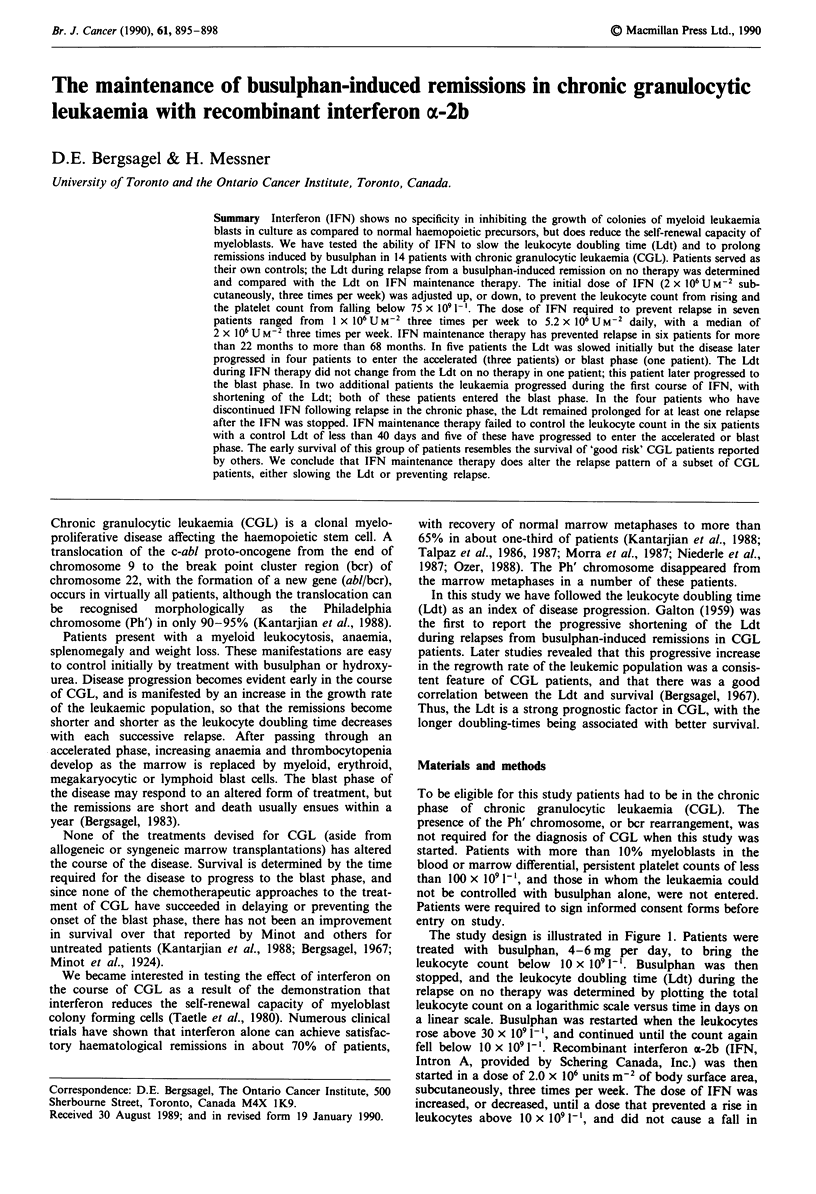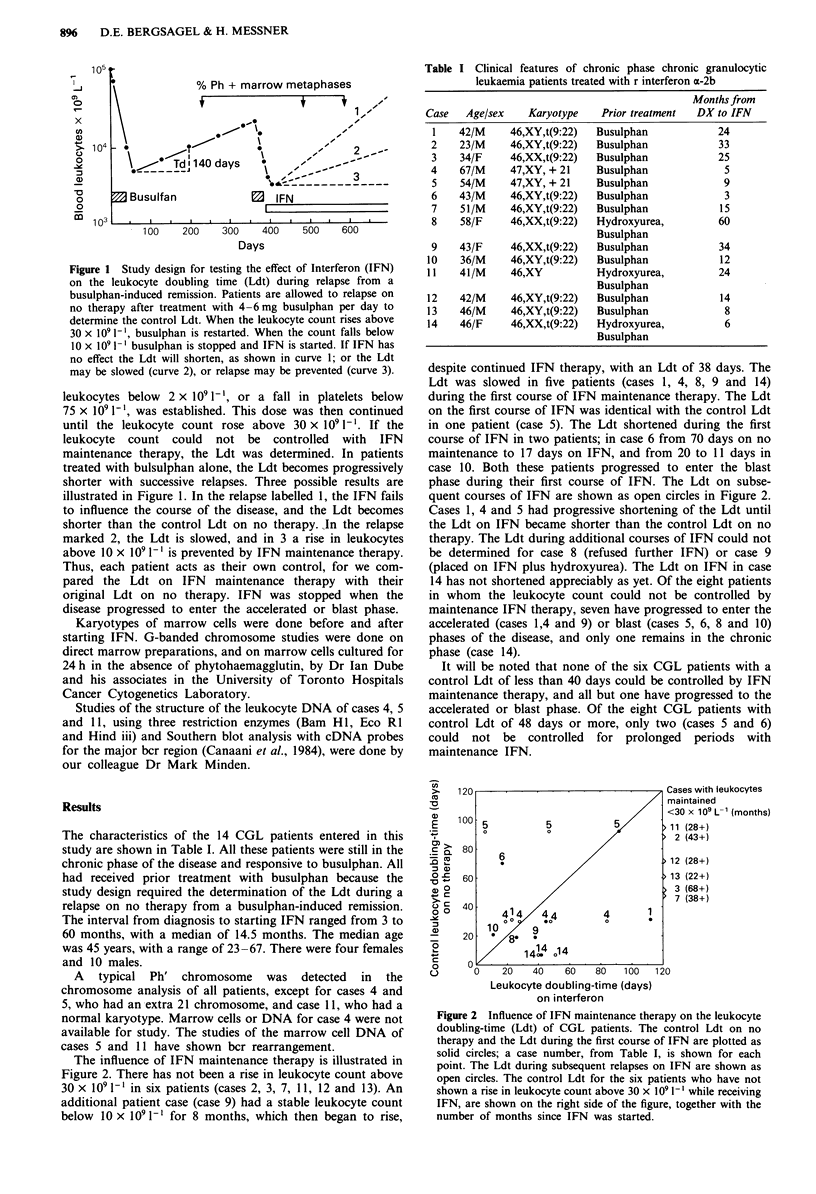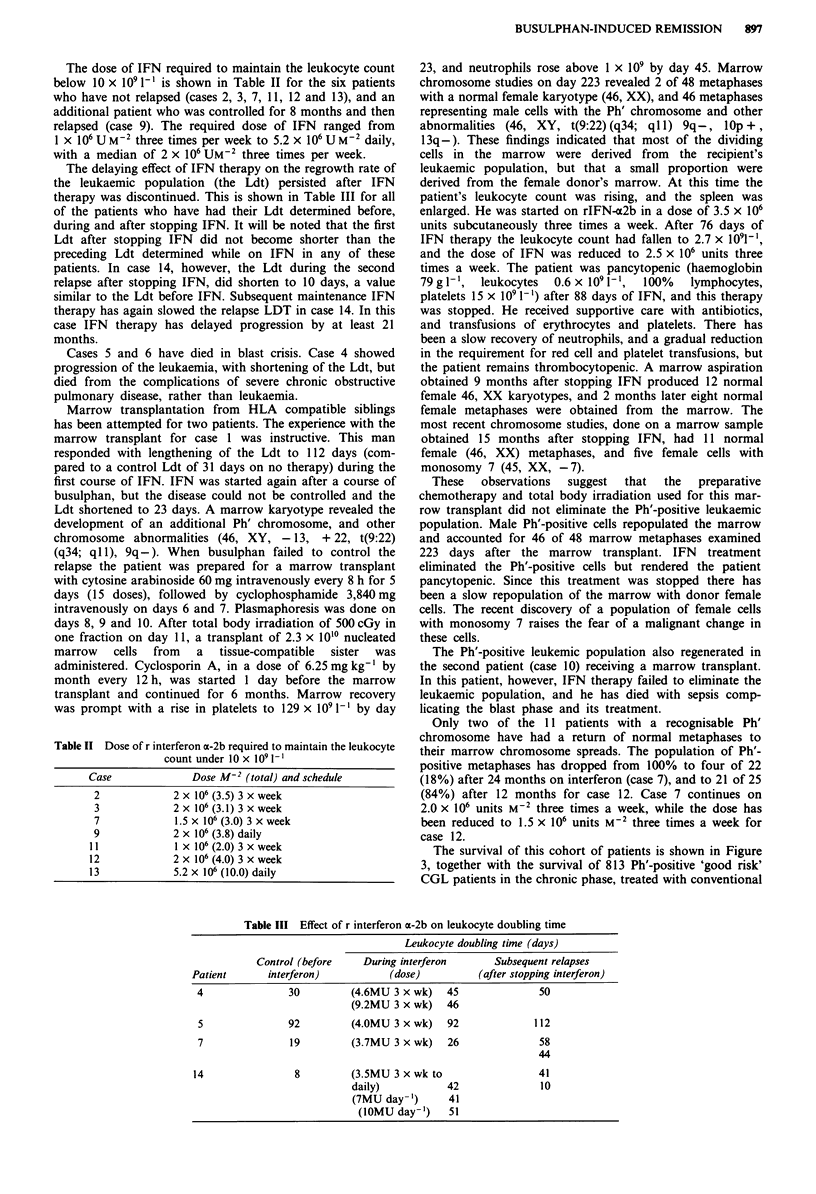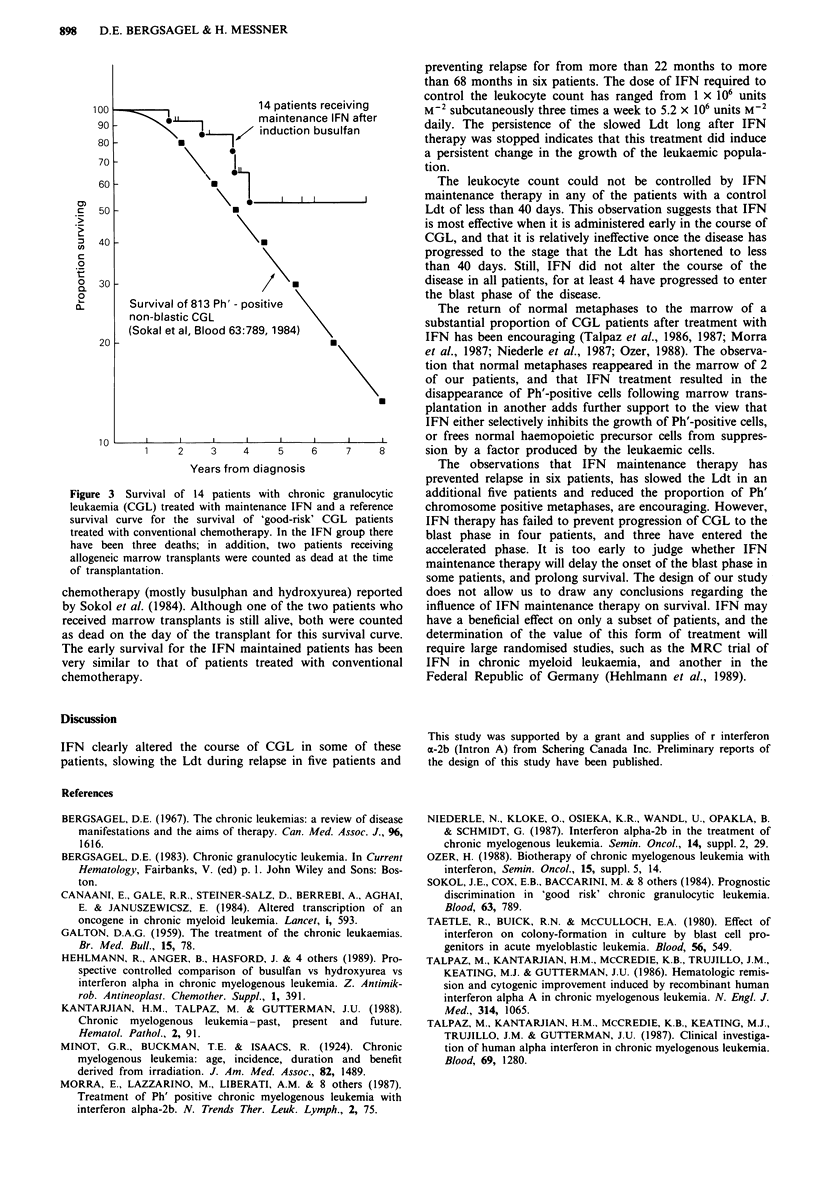# The maintenance of busulphan-induced remissions in chronic granulocytic leukaemia with recombinant interferon alpha-2b.

**DOI:** 10.1038/bjc.1990.200

**Published:** 1990-06

**Authors:** D. E. Bergsagel, H. Messner

**Affiliations:** University of Toronto, Canada.

## Abstract

Interferon (IFN) shows no specificity in inhibiting the growth of colonies of myeloid leukaemia blasts in culture as compared to normal haemopoietic precursors, but does reduce the self-renewal capacity of myeloblasts. We have tested the ability of IFN to slow the leukocyte doubling time (Ldt) and to prolong remissions induced by busulphan in 14 patients with chronic granulocytic leukaemia (CGL). Patients served as their own controls; the Ldt during relapse from a busulphan-induced remission on no therapy was determined and compared with the Ldt on IFN maintenance therapy. The initial dose of IFN (2 x 10(6) U M-2 subcutaneously, three times per week) was adjusted up, or down, to prevent the leukocyte count from rising and the platelet count from falling below 75 x 10(9) l-1. The dose of IFN required to prevent relapse in seven patients ranged from 1 x 10(6) U M-2 three times per week to 5.2 x 10(6) U M-2 daily, with a median of 2 x 10(6) U M-2 three times per week. IFN maintenance therapy has prevented relapse in six patients for more than 22 months to more than 68 months. In five patients the Ldt was slowed initially but the disease later progressed in four patients to enter the accelerated (three patients) or blast phase (one patient). The Ldt during IFN therapy did not change from the Ldt on no therapy in one patient; this patient later progressed to the blast phase. In two additional patients the leukaemia progressed during the first course of IFN, with shortening of the Ldt; both of these patients entered the blast phase. In the four patients who have discontinued IFN following relapse in the chronic phase, the Ldt remained prolonged for at least one relapse after the IFN was stopped. IFN maintenance therapy failed to control the leukocyte count in the six patients with a control Ldt of less than 40 days and five of these have progressed to enter the accelerated or blast phase. The early survival of this group of patients resembles the survival of 'good risk' CGL patients reported by others. We conclude that IFN maintenance therapy does alter the relapse pattern of a subset of CGL patients, either slowing the Ldt or preventing relapse.


### The maintenance of busulphan-induced remissions in chronic granulocytic leukaemia with recombinant interferon α-2b

D.E. Bergsagel & H. Messner

*University of Toronto and the Ontario Cancer Institute, Toronto, Canada.*

**Summary** Interferon (IFN) shows no specificity in inhibiting the growth of colonies of myeloid leukaemia blasts in culture as compared to normal haemopoietic precursors, but does reduce the self-renewal capacity of myeloblasts. We have tested the ability of IFN to slow the leukocyte doubling time (Ldt) and to prolong remissions induced by busulphan in 14 patients with chronic granulocytic leukaemia (CGL). Patients served as their own controls; the Ldt during relapse from a busulphan-induced remission on no therapy was determined and compared with the Ldt on IFN maintenance therapy. The initial dose of IFN ($2 \times 10^6$ U $M^{-2}$ subcutaneously, three times per week) was adjusted up, or down, to prevent the leukocyte count from rising and the platelet count from falling below $75 \times 10^9$ $l^{-1}$. The dose of IFN required to prevent relapse in seven patients ranged from $1 \times 10^6$ U $M^{-2}$ three times per week to $5.2 \times 10^6$ U $M^{-2}$ daily, with a median of $2 \times 10^6$ U $M^{-2}$ three times per week. IFN maintenance therapy has prevented relapse in six patients for more than 22 months to more than 68 months. In five patients the Ldt was slowed initially but the disease later progressed in four patients to enter the accelerated (three patients) or blast phase (one patient). The Ldt during IFN therapy did not change from the Ldt on no therapy in one patient; this patient later progressed to the blast phase. In two additional patients the leukaemia progressed during the first course of IFN, with shortening of the Ldt; both of these patients entered the blast phase. In the four patients who have discontinued IFN following relapse in the chronic phase, the Ldt remained prolonged for at least one relapse after the IFN was stopped. IFN maintenance therapy failed to control the leukocyte count in the six patients with a control Ldt of less than 40 days and five of these have progressed to enter the accelerated or blast phase. The early survival of this group of patients resembles the survival of 'good risk' CGL patients reported by others. We conclude that IFN maintenance therapy does alter the relapse pattern of a subset of CGL patients, either slowing the Ldt or preventing relapse.

Chronic granulocytic leukaemia (CGL) is a clonal myeloproliferative disease affecting the haemopoietic stem cell. A translocation of the c-*abl* proto-oncogene from the end of chromosome 9 to the break point cluster region (bcr) of chromosome 22, with the formation of a new gene (*abl*/bcr), occurs in virtually all patients, although the translocation can be recognised morphologically as the Philadelphia chromosome (Ph') in only 90–95% (Kantarjian *et al.*, 1988).

Patients present with a myeloid leukocytosis, anaemia, splenomegaly and weight loss. These manifestations are easy to control initially by treatment with busulphan or hydroxyurea. Disease progression becomes evident early in the course of CGL, and is manifested by an increase in the growth rate of the leukaemic population, so that the remissions become shorter and shorter as the leukocyte doubling time decreases with each successive relapse. After passing through an accelerated phase, increasing anaemia and thrombocytopenia develop as the marrow is replaced by myeloid, erythroid, megakaryocytic or lymphoid blast cells. The blast phase of the disease may respond to an altered form of treatment, but the remissions are short and death usually ensues within a year (Bergsagel, 1983).

None of the treatments devised for CGL (aside from allogeneic or syngeneic marrow transplantations) has altered the course of the disease. Survival is determined by the time required for the disease to progress to the blast phase, and since none of the chemotherapeutic approaches to the treatment of CGL have succeeded in delaying or preventing the onset of the blast phase, there has not been an improvement in survival over that reported by Minot and others for untreated patients (Kantarjian *et al.*, 1988; Bergsagel, 1967; Minot *et al.*, 1924).

We became interested in testing the effect of interferon on the course of CGL as a result of the demonstration that interferon reduces the self-renewal capacity of myeloblast colony forming cells (Taetle *et al.*, 1980). Numerous clinical trials have shown that interferon alone can achieve satisfactory haematological remissions in about 70% of patients, with recovery of normal marrow metaphases to more than 65% in about one-third of patients (Kantarjian *et al.*, 1988; Talpaz *et al.*, 1986, 1987; Morra *et al.*, 1987; Niederle *et al.*, 1987; Ozer, 1988). The Ph' chromosome disappeared from the marrow metaphases in a number of these patients.

In this study we have followed the leukocyte doubling time (Ldt) as an index of disease progression. Galton (1959) was the first to report the progressive shortening of the Ldt during relapses from busulphan-induced remissions in CGL patients. Later studies revealed that this progressive increase in the regrowth rate of the leukemic population was a consistent feature of CGL patients, and that there was a good correlation between the Ldt and survival (Bergsagel, 1967). Thus, the Ldt is a strong prognostic factor in CGL, with the longer doubling-times being associated with better survival.

### Materials and methods

To be eligible for this study patients had to be in the chronic phase of chronic granulocytic leukaemia (CGL). The presence of the Ph' chromosome, or bcr rearrangement, was not required for the diagnosis of CGL when this study was started. Patients with more than 10% myeloblasts in the blood or marrow differential, persistent platelet counts of less than $100 \times 10^9$ $l^{-1}$, and those in whom the leukaemia could not be controlled with busulphan alone, were not entered. Patients were required to sign informed consent forms before entry on study.

The study design is illustrated in Figure 1. Patients were treated with busulphan, 4–6 mg per day, to bring the leukocyte count below $10 \times 10^9$ $l^{-1}$. Busulphan was then stopped, and the leukocyte doubling time (Ldt) during the relapse on no therapy was determined by plotting the total leukocyte count on a logarithmic scale versus time in days on a linear scale. Busulphan was restarted when the leukocytes rose above $30 \times 10^9$ $l^{-1}$, and continued until the count again fell below $10 \times 10^9$ $l^{-1}$. Recombinant interferon α-2b (IFN, Intron A, provided by Schering Canada, Inc.) was then started in a dose of $2.0 \times 10^6$ units $m^{-2}$ of body surface area, subcutaneously, three times per week. The dose of IFN was increased, or decreased, until a dose that prevented a rise in leukocytes above $10 \times 10^9$ $l^{-1}$, and did not cause a fall in

Correspondence: D.E. Bergsagel, The Ontario Cancer Institute, 500 Sherbourne Street, Toronto, Canada M4X 1K9.
Received 30 August 1989; and in revised form 19 January 1990.

Figure 1 Study design for testing the effect of Interferon (IFN) on the leukocyte doubling time (Ldt) during relapse from a busulphan-induced remission. Patients are allowed to relapse on no therapy after treatment with 4–6 mg busulphan per day to determine the control Ldt. When the leukocyte count rises above $30 \times 10^9 l^{-1}$, busulphan is restarted. When the count falls below $10 \times 10^9 l^{-1}$ busulphan is stopped and IFN is started. If IFN has no effect the Ldt will shorten, as shown in curve 1; or the Ldt may be slowed (curve 2), or relapse may be prevented (curve 3).

leukocytes below $2 \times 10^9 l^{-1}$, or a fall in platelets below $75 \times 10^9 l^{-1}$, was established. This dose was then continued until the leukocyte count rose above $30 \times 10^9 l^{-1}$. If the leukocyte count could not be controlled with IFN maintenance therapy, the Ldt was determined. In patients treated with bulsulphan alone, the Ldt becomes progressively shorter with successive relapses. Three possible results are illustrated in Figure 1. In the relapse labelled 1, the IFN fails to influence the course of the disease, and the Ldt becomes shorter than the control Ldt on no therapy. In the relapse marked 2, the Ldt is slowed, and in 3 a rise in leukocytes above $10 \times 10^9 l^{-1}$ is prevented by IFN maintenance therapy. Thus, each patient acts as their own control, for we compared the Ldt on IFN maintenance therapy with their original Ldt on no therapy. IFN was stopped when the disease progressed to enter the accelerated or blast phase.

Karyotypes of marrow cells were done before and after starting IFN. G-banded chromosome studies were done on direct marrow preparations, and on marrow cells cultured for 24 h in the absence of phytohaemagglutin, by Dr Ian Dube and his associates in the University of Toronto Hospitals Cancer Cytogenetics Laboratory.

Studies of the structure of the leukocyte DNA of cases 4, 5 and 11, using three restriction enzymes (Bam H1, Eco R1 and Hind iii) and Southern blot analysis with cDNA probes for the major bcr region (Canaani *et al.*, 1984), were done by our colleague Dr Mark Minden.

### Results

The characteristics of the 14 CGL patients entered in this study are shown in Table I. All these patients were still in the chronic phase of the disease and responsive to busulphan. All had received prior treatment with busulphan because the study design required the determination of the Ldt during a relapse on no therapy from a busulphan-induced remission. The interval from diagnosis to starting IFN ranged from 3 to 60 months, with a median of 14.5 months. The median age was 45 years, with a range of 23–67. There were four females and 10 males.

A typical Ph' chromosome was detected in the chromosome analysis of all patients, except for cases 4 and 5, who had an extra 21 chromosome, and case 11, who had a normal karyotype. Marrow cells or DNA for case 4 were not available for study. The studies of the marrow cell DNA of cases 5 and 11 have shown bcr rearrangement.

The influence of IFN maintenance therapy is illustrated in Figure 2. There has not been a rise in leukocyte count above $30 \times 10^9 l^{-1}$ in six patients (cases 2, 3, 7, 11, 12 and 13). An additional patient case (case 9) had a stable leukocyte count below $10 \times 10^9 l^{-1}$ for 8 months, which then began to rise,

**Table I** Clinical features of chronic phase chronic granulocytic leukaemia patients treated with r interferon α-2b

| *Case* | *Age/sex* | *Karyotype* | *Prior treatment* | *Months from DX to IFN* |
|---|---|---|---|---|
| 1 | 42/M | 46,XY,t(9:22) | Busulphan | 24 |
| 2 | 23/M | 46,XY,t(9:22) | Busulphan | 33 |
| 3 | 34/F | 46,XX,t(9:22) | Busulphan | 25 |
| 4 | 67/M | 47,XY, + 21 | Busulphan | 5 |
| 5 | 54/M | 47,XY, + 21 | Busulphan | 9 |
| 6 | 43/M | 46,XY,t(9:22) | Busulphan | 3 |
| 7 | 51/M | 46,XY,t(9:22) | Busulphan | 15 |
| 8 | 58/F | 46,XX,t(9:22) | Hydroxyurea, Busulphan | 60 |
| 9 | 43/F | 46,XX,t(9:22) | Busulphan | 34 |
| 10 | 36/M | 46,XY,t(9:22) | Busulphan | 12 |
| 11 | 41/M | 46,XY | Hydroxyurea, Busulphan | 24 |
| 12 | 42/M | 46,XY,t(9:22) | Busulphan | 14 |
| 13 | 46/M | 46,XY,t(9:22) | Busulphan | 8 |
| 14 | 46/F | 46,XX,t(9:22) | Hydroxyurea, Busulphan | 6 |

despite continued IFN therapy, with an Ldt of 38 days. The Ldt was slowed in five patients (cases 1, 4, 8, 9 and 14) during the first course of IFN maintenance therapy. The Ldt on the first course of IFN was identical with the control Ldt in one patient (case 5). The Ldt shortened during the first course of IFN in two patients; in case 6 from 70 days on no maintenance to 17 days on IFN, and from 20 to 11 days in case 10. Both these patients progressed to enter the blast phase during their first course of IFN. The Ldt on subsequent courses of IFN are shown as open circles in Figure 2. Cases 1, 4 and 5 had progressive shortening of the Ldt until the Ldt on IFN became shorter than the control Ldt on no therapy. The Ldt during additional courses of IFN could not be determined for case 8 (refused further IFN) or case 9 (placed on IFN plus hydroxyurea). The Ldt on IFN in case 14 has not shortened appreciably as yet. Of the eight patients in whom the leukocyte count could not be controlled by maintenance IFN therapy, seven have progressed to enter the accelerated (cases 1,4 and 9) or blast (cases 5, 6, 8 and 10) phases of the disease, and only one remains in the chronic phase (case 14).

It will be noted that none of the six CGL patients with a control Ldt of less than 40 days could be controlled by IFN maintenance therapy, and all but one have progressed to the accelerated or blast phase. Of the eight CGL patients with control Ldt of 48 days or more, only two (cases 5 and 6) could not be controlled for prolonged periods with maintenance IFN.

Figure 2 Influence of IFN maintenance therapy on the leukocyte doubling-time (Ldt) of CGL patients. The control Ldt on no therapy and the Ldt during the first course of IFN are plotted as solid circles; a case number, from Table I, is shown for each point. The Ldt during subsequent relapses on IFN are shown as open circles. The control Ldt for the six patients who have not shown a rise in leukocyte count above $30 \times 10^9 l^{-1}$ while receiving IFN, are shown on the right side of the figure, together with the number of months since IFN was started.

The dose of IFN required to maintain the leukocyte count below $10 \times 10^9\,l^{-1}$ is shown in Table II for the six patients who have not relapsed (cases 2, 3, 7, 11, 12 and 13), and an additional patient who was controlled for 8 months and then relapsed (case 9). The required dose of IFN ranged from $1 \times 10^6\,U\,M^{-2}$ three times per week to $5.2 \times 10^6\,U\,M^{-2}$ daily, with a median of $2 \times 10^6\,UM^{-2}$ three times per week.

The delaying effect of IFN therapy on the regrowth rate of the leukaemic population (the Ldt) persisted after IFN therapy was discontinued. This is shown in Table III for all of the patients who have had their Ldt determined before, during and after stopping IFN. It will be noted that the first Ldt after stopping IFN did not become shorter than the preceding Ldt determined while on IFN in any of these patients. In case 14, however, the Ldt during the second relapse after stopping IFN, did shorten to 10 days, a value similar to the Ldt before IFN. Subsequent maintenance IFN therapy has again slowed the relapse LDT in case 14. In this case IFN therapy has delayed progression by at least 21 months.

Cases 5 and 6 have died in blast crisis. Case 4 showed progression of the leukaemia, with shortening of the Ldt, but died from the complications of severe chronic obstructive pulmonary disease, rather than leukaemia.

Marrow transplantation from HLA compatible siblings has been attempted for two patients. The experience with the marrow transplant for case 1 was instructive. This man responded with lengthening of the Ldt to 112 days (compared to a control Ldt of 31 days on no therapy) during the first course of IFN. IFN was started again after a course of busulphan, but the disease could not be controlled and the Ldt shortened to 23 days. A marrow karyotype revealed the development of an additional Ph′ chromosome, and other chromosome abnormalities (46, XY, −13, +22, t(9:22) (q34; q11), 9q−). When busulphan failed to control the relapse the patient was prepared for a marrow transplant with cytosine arabinoside 60 mg intravenously every 8 h for 5 days (15 doses), followed by cyclophosphamide 3,840 mg intravenously on days 6 and 7. Plasmaphoresis was done on days 8, 9 and 10. After total body irradiation of 500 cGy in one fraction on day 11, a transplant of $2.3 \times 10^{10}$ nucleated marrow cells from a tissue-compatible sister was administered. Cyclosporin A, in a dose of $6.25\,mg\,kg^{-1}$ by month every 12 h, was started 1 day before the marrow transplant and continued for 6 months. Marrow recovery was prompt with a rise in platelets to $129 \times 10^9\,l^{-1}$ by day 23, and neutrophils rose above $1 \times 10^9$ by day 45. Marrow chromosome studies on day 223 revealed 2 of 48 metaphases with a normal female karyotype (46, XX), and 46 metaphases representing male cells with the Ph′ chromosome and other abnormalities (46, XY, t(9:22)(q34; q11) 9q−, 10p+, 13q−). These findings indicated that most of the dividing cells in the marrow were derived from the recipient's leukaemic population, but that a small proportion were derived from the female donor's marrow. At this time the patient's leukocyte count was rising, and the spleen was enlarged. He was started on rIFN-α2b in a dose of $3.5 \times 10^6$ units subcutaneously three times a week. After 76 days of IFN therapy the leukocyte count had fallen to $2.7 \times 10^9 l^{-1}$, and the dose of IFN was reduced to $2.5 \times 10^6$ units three times a week. The patient was pancytopenic (haemoglobin $79\,g\,l^{-1}$, leukocytes $0.6 \times 10^9\,l^{-1}$, 100% lymphocytes, platelets $15 \times 10^9\,l^{-1}$) after 88 days of IFN, and this therapy was stopped. He received supportive care with antibiotics, and transfusions of erythrocytes and platelets. There has been a slow recovery of neutrophils, and a gradual reduction in the requirement for red cell and platelet transfusions, but the patient remains thrombocytopenic. A marrow aspiration obtained 9 months after stopping IFN produced 12 normal female 46, XX karyotypes, and 2 months later eight normal female metaphases were obtained from the marrow. The most recent chromosome studies, done on a marrow sample obtained 15 months after stopping IFN, had 11 normal female (46, XX) metaphases, and five female cells with monosomy 7 (45, XX, −7).

These observations suggest that the preparative chemotherapy and total body irradiation used for this marrow transplant did not eliminate the Ph′-positive leukaemic population. Male Ph′-positive cells repopulated the marrow and accounted for 46 of 48 marrow metaphases examined 223 days after the marrow transplant. IFN treatment eliminated the Ph′-positive cells but rendered the patient pancytopenic. Since this treatment was stopped there has been a slow repopulation of the marrow with donor female cells. The recent discovery of a population of female cells with monosomy 7 raises the fear of a malignant change in these cells.

The Ph′-positive leukemic population also regenerated in the second patient (case 10) receiving a marrow transplant. In this patient, however, IFN therapy failed to eliminate the leukaemic population, and he has died with sepsis complicating the blast phase and its treatment.

Only two of the 11 patients with a recognisable Ph′ chromosome have had a return of normal metaphases to their marrow chromosome spreads. The population of Ph′-positive metaphases has dropped from 100% to four of 22 (18%) after 24 months on interferon (case 7), and to 21 of 25 (84%) after 12 months for case 12. Case 7 continues on $2.0 \times 10^6$ units $M^{-2}$ three times a week, while the dose has been reduced to $1.5 \times 10^6$ units $M^{-2}$ three times a week for case 12.

The survival of this cohort of patients is shown in Figure 3, together with the survival of 813 Ph′-positive 'good risk' CGL patients in the chronic phase, treated with conventional

**Table II** Dose of r interferon α-2b required to maintain the leukocyte count under $10 \times 10^9\,l^{-1}$

| *Case* | *Dose $M^{-2}$ (total) and schedule* |
|---|---|
| 2 | $2 \times 10^6$ (3.5) 3 × week |
| 3 | $2 \times 10^6$ (3.1) 3 × week |
| 7 | $1.5 \times 10^6$ (3.0) 3 × week |
| 9 | $2 \times 10^6$ (3.8) daily |
| 11 | $1 \times 10^6$ (2.0) 3 × week |
| 12 | $2 \times 10^6$ (4.0) 3 × week |
| 13 | $5.2 \times 10^6$ (10.0) daily |

**Table III** Effect of r interferon α-2b on leukocyte doubling time

| | *Leukocyte doubling time (days)* | | | |
|---|---|---|---|---|
| *Patient* | *Control (before interferon)* | *During interferon (dose)* | | *Subsequent relapses (after stopping interferon)* |
| 4 | 30 | (4.6MU 3 × wk) | 45 | 50 |
| | | (9.2MU 3 × wk) | 46 | |
| 5 | 92 | (4.0MU 3 × wk) | 92 | 112 |
| 7 | 19 | (3.7MU 3 × wk) | 26 | 58 |
| | | | | 44 |
| 14 | 8 | (3.5MU 3 × wk to | | 41 |
| | | daily) | 42 | 10 |
| | | (7MU $day^{-1}$) | 41 | |
| | | (10MU $day^{-1}$) | 51 | |

**Figure 3** Survival of 14 patients with chronic granulocytic leukaemia (CGL) treated with maintenance IFN and a reference survival curve for the survival of 'good-risk' CGL patients treated with conventional chemotherapy. In the IFN group there have been three deaths; in addition, two patients receiving allogeneic marrow transplants were counted as dead at the time of transplantation.

chemotherapy (mostly busulphan and hydroxyurea) reported by Sokol *et al.* (1984). Although one of the two patients who received marrow transplants is still alive, both were counted as dead on the day of the transplant for this survival curve. The early survival for the IFN maintained patients has been very similar to that of patients treated with conventional chemotherapy.

### Discussion

IFN clearly altered the course of CGL in some of these patients, slowing the Ldt during relapse in five patients and preventing relapse for from more than 22 months to more than 68 months in six patients. The dose of IFN required to control the leukocyte count has ranged from $1 \times 10^6$ units $M^{-2}$ subcutaneously three times a week to $5.2 \times 10^6$ units $M^{-2}$ daily. The persistence of the slowed Ldt long after IFN therapy was stopped indicates that this treatment did induce a persistent change in the growth of the leukaemic population.

The leukocyte count could not be controlled by IFN maintenance therapy in any of the patients with a control Ldt of less than 40 days. This observation suggests that IFN is most effective when it is administered early in the course of CGL, and that it is relatively ineffective once the disease has progressed to the stage that the Ldt has shortened to less than 40 days. Still, IFN did not alter the course of the disease in all patients, for at least 4 have progressed to enter the blast phase of the disease.

The return of normal metaphases to the marrow of a substantial proportion of CGL patients after treatment with IFN has been encouraging (Talpaz *et al.*, 1986, 1987; Morra *et al.*, 1987; Niederle *et al.*, 1987; Ozer, 1988). The observation that normal metaphases reappeared in the marrow of 2 of our patients, and that IFN treatment resulted in the disappearance of Ph'-positive cells following marrow transplantation in another adds further support to the view that IFN either selectively inhibits the growth of Ph'-positive cells, or frees normal haemopoietic precursor cells from suppression by a factor produced by the leukaemic cells.

The observations that IFN maintenance therapy has prevented relapse in six patients, has slowed the Ldt in an additional five patients and reduced the proportion of Ph' chromosome positive metaphases, are encouraging. However, IFN therapy has failed to prevent progression of CGL to the blast phase in four patients, and three have entered the accelerated phase. It is too early to judge whether IFN maintenance therapy will delay the onset of the blast phase in some patients, and prolong survival. The design of our study does not allow us to draw any conclusions regarding the influence of IFN maintenance therapy on survival. IFN may have a beneficial effect on only a subset of patients, and the determination of the value of this form of treatment will require large randomised studies, such as the MRC trial of IFN in chronic myeloid leukaemia, and another in the Federal Republic of Germany (Hehlmann *et al.*, 1989).

This study was supported by a grant and supplies of r interferon α-2b (Intron A) from Schering Canada Inc. Preliminary reports of the design of this study have been published.

### References

BERGSAGEL, D.E. (1967). The chronic leukemias: a review of disease manifestations and the aims of therapy. *Can. Med. Assoc. J.*, **96**, 1616.

BERGSAGEL, D.E. (1983). Chronic granulocytic leukemia. In *Current Hematology*, Fairbanks, V. (ed) p. 1. John Wiley and Sons: Boston.

CANAANI, E., GALE, R.R., STEINER-SALZ, D., BERREBI, A., AGHAI, E. & JANUSZEWICSZ, E. (1984). Altered transcription of an oncogene in chronic myeloid leukemia. *Lancet*, **i**, 593.

GALTON, D.A.G. (1959). The treatment of the chronic leukaemias. *Br. Med. Bull.*, **15**, 78.

HEHLMANN, R., ANGER, B., HASFORD, J. & 4 others (1989). Prospective controlled comparison of busulfan vs hydroxyurea vs interferon alpha in chronic myelogenous leukemia. *Z. Antimikrob. Antineoplast. Chemother. Suppl.*, **1**, 391.

KANTARJIAN, H.M., TALPAZ, M. & GUTTERMAN, J.U. (1988). Chronic myelogenous leukemia-past, present and future. *Hematol. Pathol.*, **2**, 91.

MINOT, G.R., BUCKMAN, T.E. & ISAACS, R. (1924). Chronic myelogenous leukemia: age, incidence, duration and benefit derived from irradiation. *J. Am. Med. Assoc.*, **82**, 1489.

MORRA, E., LAZZARINO, M., LIBERATI, A.M. & 8 others (1987). Treatment of Ph' positive chronic myelogenous leukemia with interferon alpha-2b. *N. Trends Ther. Leuk. Lymph.*, **2**, 75.

NIEDERLE, N., KLOKE, O., OSIEKA, K.R., WANDL, U., OPAKLA, B. & SCHMIDT, G. (1987). Interferon alpha-2b in the treatment of chronic myelogenous leukemia. *Semin. Oncol.*, **14**, suppl. 2, 29.

OZER, H. (1988). Biotherapy of chronic myelogenous leukemia with interferon, *Semin. Oncol.*, **15**, suppl. 5, 14.

SOKOL, J.E., COX, E.B., BACCARINI, M. & 8 others (1984). Prognostic discrimination in 'good risk' chronic granulocytic leukemia. *Blood*, **63**, 789.

TAETLE, R., BUICK, R.N. & MCCULLOCH, E.A. (1980). Effect of interferon on colony-formation in culture by blast cell progenitors in acute myeloblastic leukemia. *Blood*, **56**, 549.

TALPAZ, M., KANTARJIAN, H.M., MCCREDIE, K.B., TRUJILLO, J.M., KEATING, M.J. & GUTTERMAN, J.U. (1986). Hematologic remission and cytogenic improvement induced by recombinant human interferon alpha A in chronic myelogenous leukemia. *N. Engl. J. Med.*, **314**, 1065.

TALPAZ, M., KANTARJIAN, H.M., MCCREDIE, K.B., KEATING, M.J., TRUJILLO, J.M. & GUTTERMAN, J.U. (1987). Clinical investigation of human alpha interferon in chronic myelogenous leukemia. *Blood*, **69**, 1280.